# Regional White Matter Integrity Predicts Treatment Response to Escitalopram and Memantine in Geriatric Depression: A Pilot Study

**DOI:** 10.3389/fpsyt.2020.548904

**Published:** 2020-11-17

**Authors:** Beatrix Krause-Sorio, Prabha Siddarth, Michaela M. Milillo, Roza Vlasova, Linda Ercoli, Katherine L. Narr, Helen Lavretsky

**Affiliations:** ^1^Jane and Terry Semel Institute for Neuroscience and Human Behavior, University of California, Los Angeles, Los Angeles, CA, United States; ^2^Department of Neurology, University of California, Los Angeles, Los Angeles, CA, United States

**Keywords:** geriatric depression, cognitive decline, memantine, magnetic resonance imaging, diffusion weighted imaging, white matter integrity, fractional anisotropy, treatment response

## Abstract

**Background:** Geriatric depression with subjective memory complaints increases the risk for Alzheimer's Disease. Memantine, a neuroprotective drug, can improve depression and help prevent cognitive decline. In our 6-months clinical trial, escitalopram/memantine (ESC/MEM) improved mood and cognition compared to escitalopram/placebo treatment (ESC/PBO; NCT01902004). In this report, we investigated whether baseline brain white matter integrity in fronto-limbic-striatal tracts can predict clinical outcomes using fractional anisotropy (FA).

**Methods:** Thirty-eight older depressed adults (mean age = 70.6, SD = 7.2) were randomized to ESC/MEM or ESC/PBO and underwent diffusion-weighted imaging (DWI) at 3 Tesla at baseline. Mood was assessed using the Hamilton Depression Rating Scale (HAMD), apathy using the Apathy Evaluation Scale (AES) and anxiety using the Hamilton Anxiety Scale (HAMA) at baseline and 6-months follow-up. FA was extracted from seven tracts of interest (six in each hemisphere and one commissural tract) associated with geriatric depression. Non-parametric General Linear Models were used to examine group differences in the association between FA and symptom improvement, controlling for age, sex, baseline symptom scores and scanner model, correcting for false discovery rate (FDR). *Post-hoc* tests further investigated group differences in axial, mean and radial diffusivity (AD, MD, and RD, respectively). Lastly, we performed an exploratory whole-brain model to test whether FA might be related to treatment response with memantine.

**Results:** There were no differences in remission rates or HAMD change between groups. In bilateral anterior and posterior internal capsule tracts and bilateral inferior and right superior fronto-occipital (IFO and SFO) fasciculus, higher FA was associated with larger improvements in depressive symptoms for ESC/MEM, but not ESC/PBO, correcting for FDR. Lower MD in the left IFO and RD in the right anterior internal capsule were associated with improved treatment responses. We found no significant associations in the whole-brain analysis.

**Limitations:** Included small sample size and high dropout.

**Conclusions:** Higher baseline FA and lower RD and MD in hypothesized fronto-limbic-striatal tracts predicted greater improvement in mood and anxiety with ESC/MEM compared to ESC/PBO in geriatric depression. FA as a biomarker for white matter integrity may serve as a predictor of treatment response but requires confirmation in larger future studies.

## Introduction

Depression is among the most common and disabling conditions in older adults (1–4). While 70% of depressed older adults successfully respond to the first-line antidepressant therapy with selective serotonin reuptake inhibitors (SSRIs) after 8–12 weeks of treatment (5), only 30–40% achieve remission (6). Geriatric depression is associated with reduced remission rates compared to younger depressed adults, which may be due to comorbid cognitive impairments with subjective memory decline and diminished executive functions (7–10). Comorbid cognitive impairments can worsen disease prognosis and increase the risk of developing Alzheimer's disease (11–13). Other frequent comorbid symptoms are anxiety and apathy, which can also moderate the antidepressant response (14, 15). Thus, it is important to develop new therapeutic approaches that target mood, anxiety, and cognitive symptoms, taking into consideration the symptom profile of geriatric depression.

Memantine is a fast-acting, well-tolerated, uncompetitive N-methyl-D-aspartate (NMDA) receptor antagonist and is FDA-approved for the treatment of Alzheimer's disease. NMDA receptors are ionotropic and bind glutamate, the major excitatory neurotransmitter in the nervous system. Increased glutamate concentrations have been detected in younger adults with major depressive disorder (MDD), and prefrontal cortex glutamate was found increased in geriatric depression (16, 17). Excessive glutamate can lead to excitotoxicity, leading to both acute neural injury and chronic neuronal degeneration (18). Therefore, memantine can be promising as an adjunct treatment for late-life depression, and potentially for the prevention of Alzheimer's disease in this high-risk population. While memantine has previously been tested for the treatment of depression, a systematic review reported contradictory findings, and a more recent meta-analysis was unable to confirm beneficial effects of memantine on mood in depression in younger populations (19–21).

In the parent double-blind randomized placebo-controlled trial (RCT) of geriatric depression with subjective memory complaints [(22), [NCT01902004]], we compared treatment response to escitalopram combined with memantine (ESC/MEM) to escitalopram combined with placebo (ESC/PBO). We did not find group differences in changes in mood at 6 or 12 months, but found improved cognition at 12-months follow-up in the ESC/MEM group. The use of brain biomarkers can address variability in treatment response and potentially identify subgroups that are more likely to respond to the targeted treatment, thus providing a more personalized approach to treatment selection. For example, we have found that higher baseline amyloid and tau markers in the frontal lobe, assessed with (2-(1-{6-[(2-[fluorine-18]fluoroethyl)(methyl)amino]-2-naphthyl}-ethylidene)) [^18^F]FDDNP positron emission tomography (PET), was associated with greater improvement in executive functions at 6 months in both treatment group, thus identifying a potential biomarker of cognitive improvement with antidepressant use (23).

Prior studies demonstrated that both gray and white matter atrophy observed in geriatric depression mostly affected fronto-limbic-striatal regions and their structural connections (24–28). This included longitudinal volume reductions in frontal and association regions in geriatric depression compared to healthy controls, as well as white matter decreases within the superior frontal gyrus, posterior thalamic radiation, corpus callosum, and the superior longitudinal fasciculus over a 2-years period (29). A systematic review from 15 existing studies supports that fractional anisotropy (FA), a marker for white matter integrity, is consistently reduced in geriatric depression compared to healthy controls in frontal and fronto-limbic tracts (30, 31). One prior study comparing FA at baseline between remitted and non-remitted older adults with depression treated with escitalopram for 12 weeks demonstrated reduced FA in fronto-limbic tracts including sub-regions of the anterior cingulate gyrus (ACC) and the posterior cingulate (PCC), the dorsolateral prefrontal cortex, the genu of the corpus callosum, and in white matter regions adjacent to the hippocampus and the insula in non-remitters compared to those that achieved remission (32). To summarize, reductions in the diffusion tensor imaging measure FA have been reported in fronto-limbic fiber pathways in geriatric depression with some consistency. However, the majority of published studies in this field have only examined cross-sectional differences in diffusion imaging measures between geriatric depression and healthy controls. Although one prior report suggests that pre-treatment FA may serve as an indicator of successful therapeutic outcome (32), associations between FA and treatment-related changes in symptoms in geriatric depression remain unaddressed. Further, how escitalopram combined with memantine interacts with white matter integrity in association with symptom improvement remains unknown.

In this exploratory study, we tested whether FA can be used prospectively as a biomarker of treatment response in geriatric depression. Specifically, we investigated whether baseline FA, a biomarker of white matter integrity measured with diffusion-weighted imaging (DWI), could predict treatment response to combined escitalopram and memantine (ESC/MEM) compared to escitalopram and placebo (ESC/PBO). To further identify the underlying structural mechanisms of the observed relationships between FA and symptom improvement, we investigated axial diffusivity (AD; reflecting axonal density), radial diffusivity (RD; myelination) and mean diffusivity (MD; magnitude of overall diffusion associated with tissue atrophy) in a *post-hoc* analysis.

## Materials and Methods

### Participants

**Figure 1:**
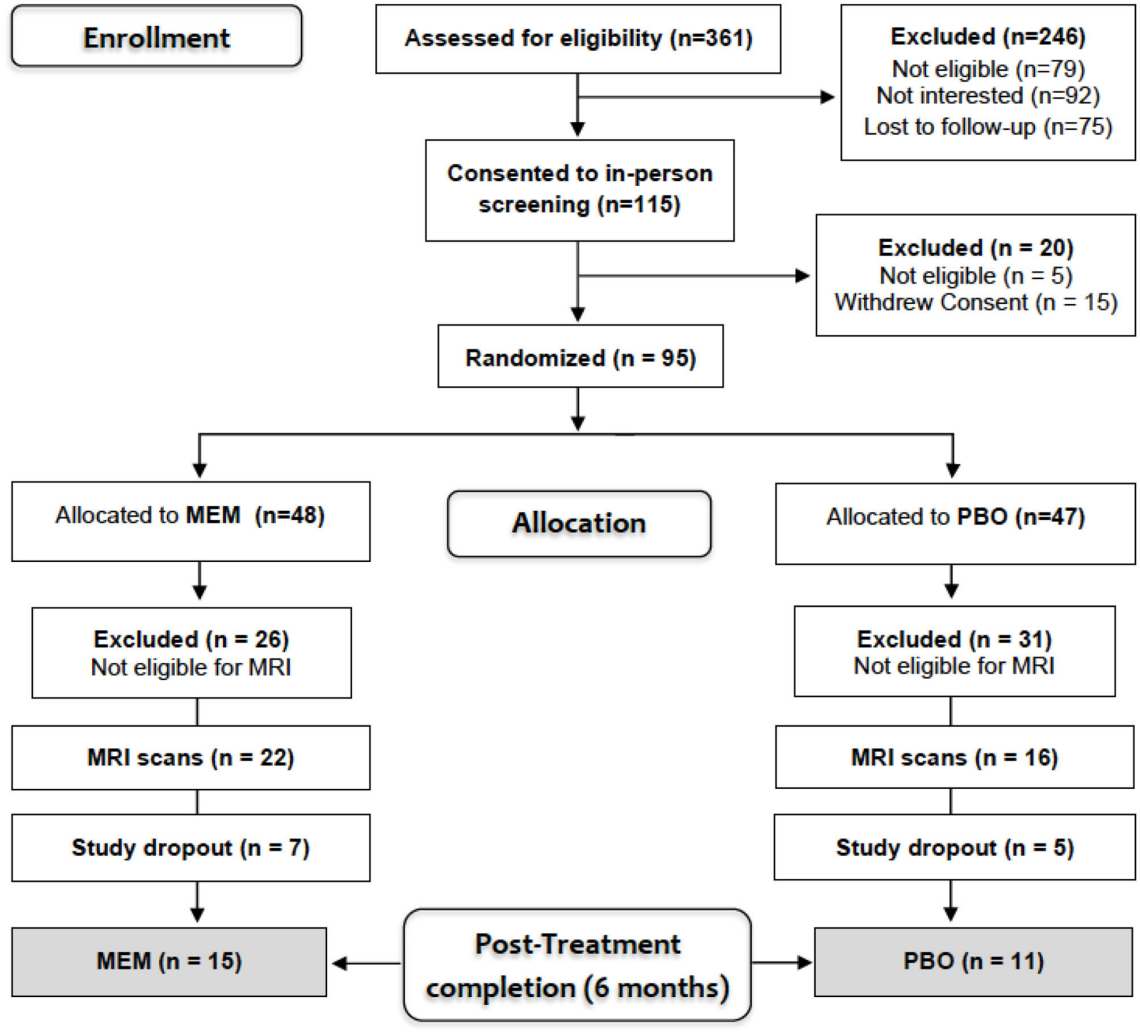
Consort diagram.

Participants included a sub-sample of 38 older adults (mean age = 70.6, SD = 7.2; 14 men/24 women) diagnosed with MDD (mean age = 70.6, SD = 7.2; 14 male/24 female) who participated in a larger clinical randomized placebo-controlled trial [RCT-NCT01902004, (22)] comparing the efficacy of ESC/MEM compared to ESC/PBO in treating geriatric depression with subjective memory complaints. Only 38 out of 95 were eligible for magnetic resonance imaging (MRI) scanning due to exclusion criteria of claustrophobia, or metallic implants that were deemed unsafe for scanning at 3 Tesla. These participants underwent diffusion-weighted imaging (DWI) at baseline (for patient characteristics, see Table 1) and based on the randomization of the parent clinical trial, 22 participants were randomized to receive ESC/MEM, while 16 received ESC/PBO (Figure 1). Only 26 participants completed MRI at follow-up and were included in this analysis. Inclusion criteria were: age ≥ 60 years; a DSM-5 (Diagnostic and Statistical Manual) diagnosis of MDD (33), and a Hamilton Rating Scale for Depression score of 16 or greater [HAMD-24; (34)], a Mini-Mental State Examination score of 23 or greater [MMSE; (35)] and a subjective report of memory impairment. Subjective memory complaints were assessed during the phone screening as an affirmative response to the question “Have you experienced memory problems over the past 6 months?” Exclusion criteria were a history of psychiatric disorders, including substance abuse disorder, suicidal behavior or suicide attempts within the past year; acute or severe current or recent medical illness; a history of allergies or intolerance to either escitalopram or memantine. The study was approved by the Institutional Review Board at the University of California Los Angeles (UCLA). Participants signed written informed consent prior to the beginning study procedures.

**Table 1:** Participant demographics and baseline clinical scores of the subsample of 38 participants used in the current study.

	**ESC/MEM Mean (standard deviation)**	**ESC/PBO Mean (standard deviation)**	***Kruskal-Wallis test statistic***	***p*-value**
	***N* = 22**	***N* = 16**		
Sex (m/f)	8/14	6/10		Fisher's exact *p =* 1.0
MCI diagnosis	5	2		Fisher's exact *p =* 0.68
Age	70.05 (7.33)	71.25 (7.15)	0.32	0.57
Education in years	15.82 (2.17)	16.13 (2.13)	0.37	0.54
MMSE	28.45 (1.53)	28.19 (1.72)	0.16	0.69
HAMD	17.64 (2.34)	17.69 (2.24)	<0.0001	1.0

*There were no significant differences between treatment groups at baseline*.

*HAMD = Hamilton Depression Rating Scale; MMSE = Mini Mental State Examination*.

### Clinical and Cognitive Assessment

Remission was defined as a HAMD score of six or lower at follow-up (36). Participants completed the Clinical Dementia Rating Scale [CDR; (37)], and the MMSE (35) at baseline to exclude those with dementia. Mild cognitive impairment (MCI) was defined as the stage between normal cognition and dementia (CDR = 0.5), subjective reports of cognitive decline as experienced by the participant or a collateral, objective neurocognitive impairment, but the absence of significant functional impairment (38). The Wechsler Memory Scale Third Edition, WMS-III, including the verbal paired associates subtest [Total or Delayed scores; (39)] and the Hopkins Verbal Learning Test [HVLT; (40)] were additionally administered. Objective neurocognitive impairment was defined as a score below at least one standard deviation (SD) of age- and education-specific norms on two or more screening memory tests: HVLT Total or Delayed memory, and Wechsler Memory Scale Third Edition, WMS-III, verbal paired associates [Total or Delayed scores; (41, 42)]. In addition, we administered the Hamilton Anxiety Scale [HAMA, (43)] and the Apathy Evaluation Scale [AES, (44)].

### Medication Administration and Adherence

During the first 4 weeks of treatment, the daily dose of escitalopram was 10 mg, while daily memantine or matched placebo doses were titrated up from 5 to 20 mg over the course of the first 4 weeks. The Clinical Global Impression Scale [CGI; (45)] was administered at baseline and follow-up to track the severity and improvement of depressive symptoms. If the CGI score was ≥3 after week four, escitalopram was increased to 20 mg. Memantine was adjusted to a minimum of 5 mg and escitalopram to 10 mg based on tolerability.

### Neuroimaging Protocol

DWI images were collected using either a 3T Siemens TIM Trio or Prisma system (Siemens, Erlangen, Germany) due to a hardware upgrade mid-study with a 32-channel head coil at the UCLA Ahmanson & Lovelace Brain Mapping Center. Consequently, 4/15 participants in the ESC/MEM and 4/11 participants in the ESC/PBO group were scanned on the Trio. For co-registration, a T1-weighted image was acquired with parameters matched across scanners: a multi-echo magnetization-prepared rapid acquisition gradient echo (MPRAGE) scan: 1 mm^3^ with isotropic voxel dimensions, 176 slices, repetition time = 2,150 ms, echo time = 1.74, 3.6, 5.46, and 7.32 ms, inversion time = 1,260, field of view (FOV) = 256 mm, matrix size = 256 × 256 mm, and a flip angle of 7 degrees. DWI parameters were: Trio: multi-band factor = 3; 72 slices; 144 gradient directions; 1.8 mm^3^ isotropic voxel size; field of view = 190 mm; repetition time = 3.245 ms; echo time = 84 ms; 12 b0's; 90-degree flip angle; b-factor = 1,000 s/mm^2^. Prisma: multi-band factor = 4; 98 gradient directions; 92 slices; 1.5 mm^3^ isotropic voxel size; field of view = 210 mm; repetition time = 3.23; echo time = 89.2 ms; 7 b0's; flip angle = 78 degrees; and b-factor = 1,500 s/mm^2^.

### DWI Data Processing

DWI images were processed using FSL software (http://fsl.fmrib.ox.ac.uk/fsl/fslwiki/) after visual inspection of gradient direction images for artifacts or excessive movement. DWI data underwent anatomical distortion correction (46), eddy-current and movement artifact correction. Further processing steps were equal for both scanner acquisition protocols: eddy-current and movement artifact correction. The gradient direction vectors (b-vectors) were adjusted accordingly. Subsequently, we separated the brain from its surrounding tissues, including cerebrospinal fluid, bone, fat and skin, and the non-brain tissue was removed from the images using the brain-extraction tool (47). A diffusion tensor model was fitted to the resulting data. Using FSL's Tract-based Spatial Statistics workflow [TBSS; (48)] individual FA maps were warped into 1 mm Montreal Neurological Institute (MNI) space and a study-specific white matter skeleton was created from all participants' images with a FA threshold of 0.2. Individual subjects' FA values were then projected onto the group white matter skeleton. This skeletonized data was used to extract mean FA values for each participant for seven tract regions-of-interest (ROIs). ROIs were derived from the ICBM-DTI-81 probabilistic white matter label atlas (https://fsl.fmrib.ox.ac.uk/fsl/fslwiki/Atlases) and comprised the cross-hemisphere genu of the corpus callosum (GCC), and the left and right anterior and posterior limb of the internal capsule (ALIC, PLIC, respectively), cingulum bundle of the cingulate gyrus (CGC), the fornix (FX, left, right and the commissural body), inferior and superior fronto-occipital (IFO, SFO) and superior longitudinal fasciculi (SLF). The same procedure was applied to extract AD, MD, and RD metrics from each tract ROI, respectively.

### Statistics

Due to the small sample size, non-parametric methods were used for all analyses (49). We used Kruskal-Wallis test to test baseline group differences in mean FA for all ROIs (six for each hemisphere plus one commissural) between Siemens Trio and Prisma scanners. For the following analyses, as recommended by Conover and Iman (50) and Conover (51), we used a rank transformation on all of the variables and then estimated standard general linear models (GLMs). Between-group differences in HAMD changes were examined with a rank-based GLM, with treatment group (ESC/MEM vs. ESC/PBO) as the predictor, controlling for the baseline score, age and sex. Similar rank-based GLMs were estimated to examine group differences in associations of HAMD change and mean FA in the ROIs of interest (L and R of ALIC, PLIC, CGC, IFO, FX, SFO, and SLF as well as FX body GCC), by including the mean FA and their interaction with treatment group as additional predictors, as well as scanner model (Trio vs. Prisma). We used the Benjamini-Hochberg procedure (with a false discovery rate of 10%) to correct for multiple comparisons. We also report Spearman rank correlation coefficients for all significant associations. As follow-up analyses, we estimated similar models on AD, MD, and RD for those tracts showing significant FA effects. While the tracts of interest comprised our main analysis, we additionally performed a GLM (group × change in HAMD on FA) exploratory voxel-wise whole skeleton analysis using 10,000 permutations and threshold-free cluster correction (TFCE) correction at an alpha level of 0.05.

## Results

### Baseline

Baseline demographics, as well as clinical scores and symptoms are detailed in Table 1 for the subset of 38 participants included in this study (also see Supplementary Table 1). The parent clinical trial included 95 participants, described in the primary article (22). The groups in this subsample also did not differ in demographic and clinical scores at baseline (Table 1). No images were excluded for motion or other artifacts and there was no difference in baseline FA averaged across ROIs between Trio and Prisma scanners [left FA: *x*^2^(1) = 0.03, *p* = 0.87; right FA: *x*^2^(1) = 3.09, *p* = 0.15].

### Treatment Effects

Of the 38 participants who underwent an MRI at baseline, 26 (15 in the ESC/MEM group and 11 in the ESC/PBO group) completed the study. The groups of completers (ESC/MEM vs. ESC/PBO) did not differ in demographic and clinical data at baseline [sex: Fisher's exact *p* = 0.7; age: Kruskal-Wallis *x*^2^(1) = 0.02, *p* = 0.9; education: *x*^2^(1) = .<0.01, *p* = 1.0; MMSE: *x*^2^(1) = 0.1.73, *p* = 0.2; HAMD: *x*^2^(1) = 0.3, *p* = 0.6]. Remission was achieved by 11/15 completers (73%) in the ESC/MEM group, and 6/11 completers (55%) in the ESC/PBO group. There was no significant difference in remission rates between treatment groups at 6 months (Fisher's exact *p* = 0.42). There was also no significant difference between groups for 6-months change in HAMD [*F*_(1, 21)_ = 0.92, *p* = 0.3; Table 2, Supplementary Table 2].

**Table 2:** HAMD scores at baseline and 6 months by treatment group for the 26 completers of the trial.

**Variable**	**ESC/MEM Mean (standard deviation)** ***N*** **=** **15**			**ESC/PBO Mean (standard deviation)** ***N*** **=** **11**			**Between-group change**	
	**Baseline**	**6 months**	**Change**	**Baseline**	**6 months**	**Change**	***F*_**(1, 21)**_ statistic**	***p*-value**
HAMD	17.6	5.67	−11	17.73	7.18	−10.55	0.92	0.3
	(2.61)	(5.72)	(6.16)	(1.95)	(5.04)	(4.23)		

*There were no significant group differences in HAMD change. The results presented here stem from the rank-based general linear models*.

*HAMD, Hamilton Depression Rating Scale*.

### Neuroimaging Results

**Figure 2:**
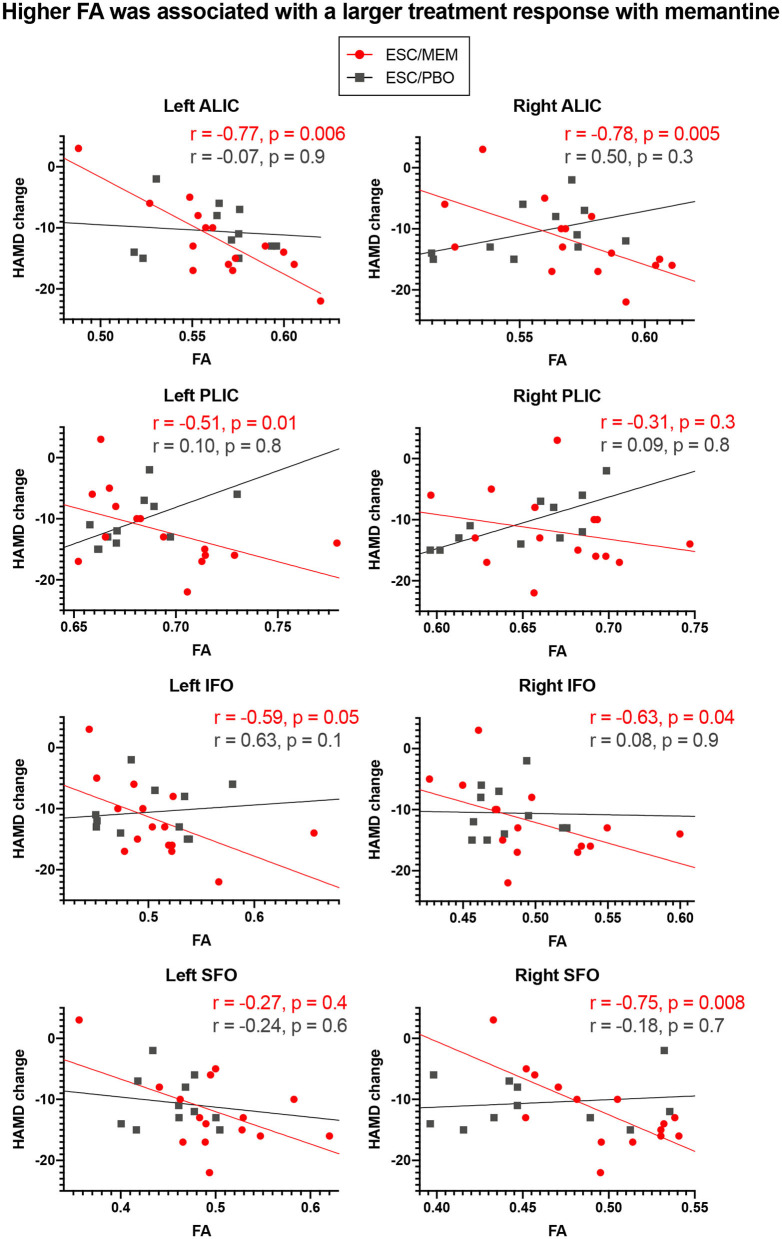
Higher baseline FA in ROIs was associated with larger clinical improvements with memantine but not placebo treatment. Higher baseline FA in regions associated with geriatric depression was associated with greater improvement in depressive symptoms in bilateral ALIC, PLIC, and IFO and the right SFO in ESC/MEM treated participants. There was no relationship between FA and clinical improvement in the placebo group. While partial Spearman correlation coefficients (controlling for age, sex, baseline HAMD score, and scanner) were used, the plots depict (non-ranked) original values for better interpretability. HAMD, Hamilton Depression Scale.

Correcting for multiple comparisons, significant group x FA interactions were obtained for baseline FA and change in HAMD in the left and right ALIC, PLIC, and IFO, as well as in the right SFO (Table 3). For all of these significant interactions, follow-up analyses revealed that higher baseline FA was associated with a larger improvement in depressive symptoms in the ESC/MEM group (Figure 2). In the right PLIC, the effect within the ESC/MEM group did not reach significance (t = −1.84, *p* = 0.08). None of the associations between baseline FA and HAMD change were significant for the ESC/PBO group. The follow-up exploratory analyses on AD and RD revealed no significant interactions in the ALIC, PLIC, IFO, and right SFO, while RD showed a similar group x RD interaction in the right ALIC (*F*_(1, 18)_ = 5.07, *p* = 0.04) which did not reach significance in the left ALIC [*F*_(1, 18)_ = 3.97, *p* = 0.06; see Supplementary Table 3]. This was due to a negative association of RD with HAMD change in the ESC/MEM (t = −3.27, *p* = 0.004) but not the ESC/PBO group (t = 0.31, *p* = 0.76). For MD, there were trends in bilateral ALIC and PLIC, the right SFO (Table 3) and a significant interaction in the left [*F*_(1, 18)_ = 0.66, *p* = 0.43], but not the right IFO [*F*_(1, 18)_ = 1.06, *p* = 0.32]. Contrary to the results for FA, higher left IFO MD was associated with a poorer improvement in depressive symptoms in the ESC/MEM (t = 2.23, *p* = 0.04), but not the ESC/PBO group (t = −1.17, *p* = 0.26). There were no significant clusters for FA, AD, MD or RD in the voxel-wise whole-brain analysis of group differences. Supplementary Table 4 and Supplementary Figure 1 show FA × ROI interactions for anxiety (HAMA) and apathy (AES).

**Table 3:** Relationship between change in HAMD and baseline FA in ESC/MEM and ESC/PBO.

**FA**	**Left**			**Right**		
	**Interaction group x FA**	**ESC+MEM**	**ESC+PBO**	**Interaction group x FA**	**ESC+MEM**	**ESC+PBO**
**HAMD**						
ALIC	*F*_(1, 18)_ = 4.66, *p =* 0.04^*^	T = −3.63, *p =* 0.002; r = −0.77	T = −0.56, *p* = 0.58	*F*_(1, 18)_= 7.7, *p =* 0.01^*^	T = −5.11, *p* ≤ 0001; r = −0.78	T = −0.76, *p =* 0.45
PLIC	*F*_(1, 18)_ = 7.71, *p =* 0.01	T = −2.73, *p =* 0.01; r = −0.51	T = 1.35, *p =* 0.19	*F*_(1, 18)_= 5.76, *p =* 0.03^*^	T = −1.84, *p =* 0.08; r = −0.31	T = 1.09, *p =* 0.29
CGC	*F*_(1, 18)_ = 0.34, *p =* 0.56			*F*_(1, 18)_= 2.11, *p =* 0.16		
IFO	*F*_(1, 18)_ = 4.65, *p =* 0.04^*^	T = −2.57, *p =* 0.02; r = −0.59	T = 0.23, *p =* 0.82	*F*_(1, 18)_ = 4.69, *p =* 0.04^*^	T = −3.58, *p =* 0.002; r = −0.63	T = 0.18, *p =* 0.86
SFO	*F*_(1, 18)_ = 0.17, *p =* 0.69			*F*_(1, 18)_= 8.48, *p =* 0.009**	T = −3.63, *p =* 0.002; r = −0.75	T = −0.04, *p =* 0.97
SLF	*F*_(1, 18)_ = 1.49, *p =* 0.24			*F*_(1, 18)_= 3.31, *p =* 0.09		
FX	*F*_(1, 18)_ = 0.24, *p =* 0.63			*F*_(1, 18)_= 0.01, *p =* 0.94		
FX Body	Interaction: *F*_(1, 18)_ = 0.06, *p =* 0.80					
GCC	Interaction: *F*_(1, 18)_ = 0.13, *p =* 0.73					

*The results stem from the rank-based general linear models including the treatment group, FA and the interaction between group and FA as the predictors, while controlling for the respective baseline score, age, sex and scanner*.

*HAMD, Hamilton Depression Scale*.

* *Significant after FDR correction*.

## Discussion

This pilot study is the first to demonstrate that higher baseline measures of brain white matter integrity may predict changes in symptoms of depression in a 6-months trial of escitalopram combined with memantine compared Toto placebo. Similar to results from the parent trial, in the current subset of participants who underwent MRI scanning, mood symptoms improved in both treatment groups (22). In bilateral ALIC, PLIC, and IFO, as well as the right SFO, components of the fronto-limbic-striatal network, baseline FA was associated with improvements in mood in the memantine, but not the placebo group. RD showed trends in the same direction in bilateral PLIC and the left ALIC, though only changes in RD in the right ALIC reached significance. This suggests that the mechanism underlying poorer treatment response to memantine in patients with higher RD may be attributed to demyelination processes (52). We did not observe any trends or effects in AD. MD, reflecting tissue atrophy rather than integrity, showed the opposite directionality of FA in the left IFO, but no effect in the right hemisphere. This further implicates that those with reduced white matter integrity in this region may respond less well to treatment with memantine. In contrast to the effects for improvement in depressive symptoms in bilateral ALIC, PLIC, IFO, and right SFO, larger improvements in anxiety were linked to higher FA in the left ALIC and SLF, as well as in bilateral CGC and IFO in the ESC/MEM but not the ESC/PBO group (Supplementary Table 4). For apathy, higher FA in the right SFO was associated with better treatment outcomes in the ESC/MEM group. While the limited sample size does not allow for broader generalizations, it appears from our results that white matter prediction effects might be symptom-, tract- and treatment-specific.

The use of non-invasive neuroimaging markers for prediction of treatment response has been previously recommended (53), further supporting the usefulness of FA in predicting the effects of combined antidepressant treatment with memantine in geriatric depression. For instance, it has been suggested that abnormalities in fronto-limbic white matter connectivity in geriatric depression plays a role in the reduced antidepressant response based on genetic factors for neuroprotective mechanisms (54). Another study on 12 weeks of escitalopram treatment found that remitters could be distinguished from non-remitters at baseline based on FA in fronto-limbic tracts including some of our selected ROIs (32). Furthermore, escitalopram effects on apathy have been found to be independent of its effects on mood in geriatric depression and could be predicted by baseline FA in the left uncinate fasciculus, a tract we did not examine in the current study (55). White matter integrity declines with aging and can affect treatment response in geriatric depression. It should be investigated whether the neuroprotective drug memantine can increase white matter connectivity in impaired circuits.

There are several limitations to the current study. First, the small sample size limited our statistical power. This study was intended as a hypothesis-generating pilot study and findings presented here must be replicated in larger longitudinal studies. Nonetheless, we demonstrated the feasibility of using MRI markers for fronto-limbic-striatal tract integrity as a possible predictor of antidepressant and cognitive enhancement treatment response in geriatric depression. The limited sample size was mainly due to contraindications to MRI scanning, such as implanted devices deemed unsafe for MRI scanning at 3 Tesla, as well as high dropout rates that might limit the generalizability of our findings. Third, we did not investigate change in FA from baseline to the 6-montha follow-up, such that we were unable to test the association between treatment-related change in clinical improvements and FA changes. Fourth, this was a secondary analysis of the primary RCT and used only a subset of the sample who completed the RCT and also had neuroimaging data. Fifth, our selection of tracts of interest was not inclusive of all major white matter pathways in the brain, but instead focused on those pathways previously implicated in geriatric depression. As reflected in the absence of results from our whole-brain analysis, we likely lack the power to detect effects at the voxel-wise level and larger studies using whole-brain models can investigate whether clusters appear to indicate more regional specificity. Lastly, it is important to note that FA has limited value as a proxy for white matter integrity, as it is highly sensitive to changes at the microstructural level (56).

In summary, in our pilot study of geriatric depression, we demonstrated the ability of baseline regional white matter tract integrity to predict treatment outcomes in a randomized placebo-controlled trial of escitalopram and memantine or placebo. While we were unable to detect significant differences in clinical response to memantine, we demonstrated that white matter health in fronto-limbic-striatal tracts was associated with symptom improvement favoring memantine. Our results suggest that even in the absence of clinical effects, FA in components of fronto-limbic-striatal tracts might be a biomarker of treatment response with memantine in older populations with depression, whereby higher indicators of white matter integrity were associated with improved treatment responses. Future studies might focus on treatment-related changes in structural and functional connectivity in larger prospective samples.

## Data Availability Statement

The raw data supporting the conclusions of this article will be made available by the authors, without undue reservation.

## Ethics Statement

The studies involving human participants were reviewed and approved by University of California Los Angeles Institutional Review Board. The patients/participants provided their written informed consent to participate in this study.

## Author Contributions

HL conceived the study, obtained funding, and led the study. PS performed the statistical analysis. LE provided and supervised the neuropsychological testing procedures. BK-S and RV performed scans and processed and analyzed the neuroimaging data. BK-S wrote the manuscript with the help of all co-authors. KN and MM assisted in all aspects. All authors were involved in working on the manuscript.

## Conflict of Interest

HL has received research grants from Allergan, NIMH, NCCIH, PCORI, and the Alzheimer's Research & Prevention Foundation. The remaining authors declare that the research was conducted in the absence of any commercial or financial relationships that could be construed as a potential conflict of interest.

## Supplementary Material

Supplementary Figure 1 Higher baseline FA was associated with larger clinical improvements with memantine but not placebo treatment on anxiety and apathy symptoms. Higher baseline FA in regions associated with geriatric depression was associated with greater improvement in anxiety symptoms in the left ALIC and SLF and bilateral CGC and IFO in ESC/MEM treated participants. In the right SFO, there was a similar effect for apathy (note that higher scores reflect weaker symptoms). There was no relationship between FA and clinical improvement in the placebo group. While partial Spearman correlation coefficients (controlling for age, sex, baseline HAMD score and scanner) were used, the plots depict (non-ranked) original values for better interpretability. HAMA = Hamilton Anxiety Scale; AES = Apathy Evaluation Scale.
