# Supplementary figures and images for: Regional White Matter Integrity Predicts Treatment Response to Escitalopram and Memantine in Geriatric Depression: A Pilot Study
Source: Front Psychiatry. 2020 Nov 17;11:548904. doi: 10.3389/fpsyt.2020.548904 (PMC7718009; doi:10.3389/fpsyt.2020.548904)

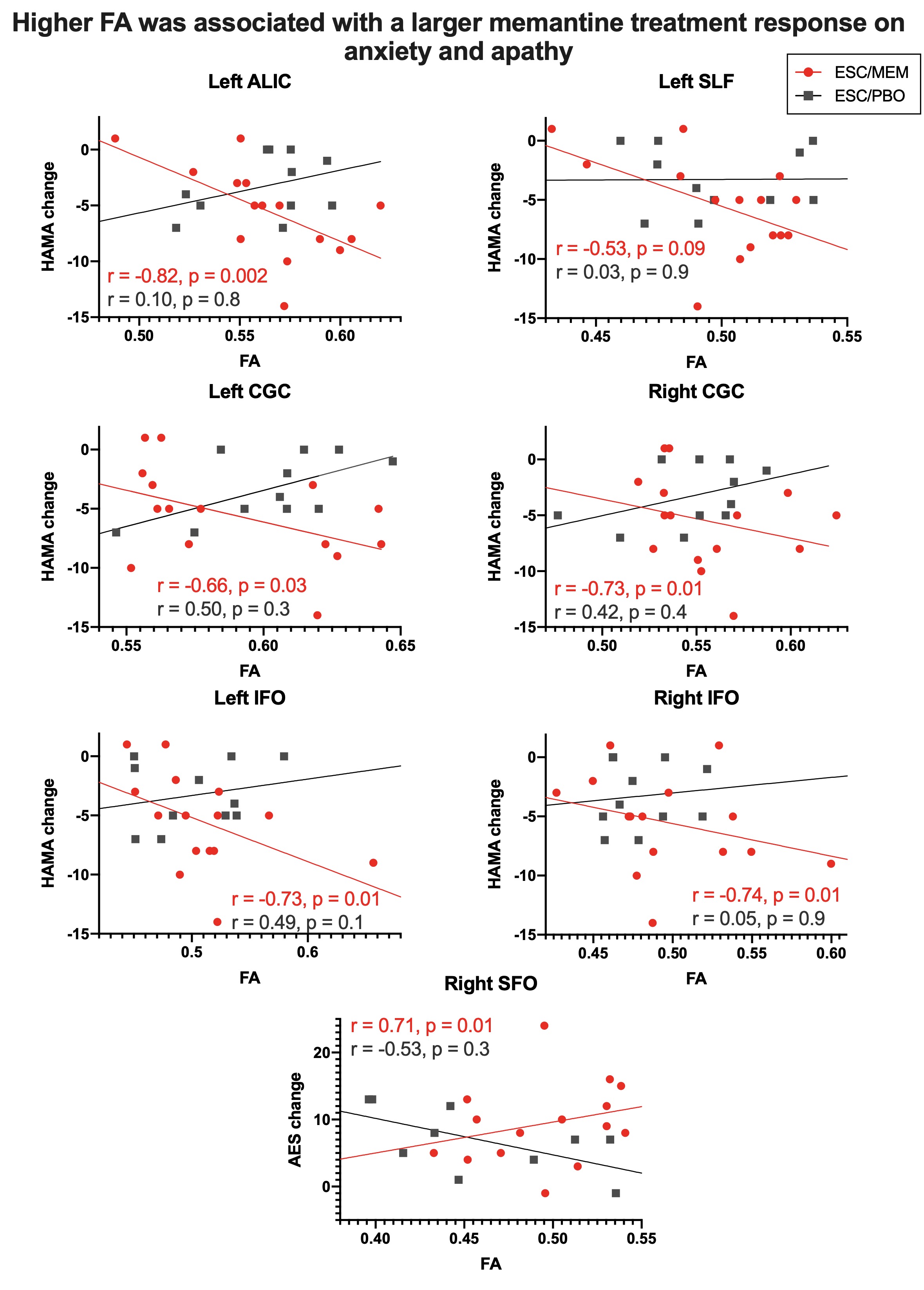

Supplement: Supplementary Figure 1 — Higher baseline FA was associated with larger clinical improvements with memantine but not placebo treatment on anxiety and apathy symptoms. Higher baseline FA in regions associated with geriatric depression was associated with greater improvement in anxiety symptoms in the left ALIC and SLF and bilateral CGC and IFO in ESC/MEM treated participants. In the right SFO, there was a similar effect for apathy (note that higher scores reflect weaker symptoms). There was no relationship between FA and clinical improvement in the placebo group. While partial Spearman correlation coefficients (controlling for age, sex, baseline HAMD score and scanner) were used, the plots depict (non-ranked) original values for better interpretability. HAMA = Hamilton Anxiety Scale; AES = Apathy Evaluation Scale. [file Image_1.jpg]
