# Supplementary material for: Regional White Matter Integrity Predicts Treatment Response to Escitalopram and Memantine in Geriatric Depression: A Pilot Study
Source: Front Psychiatry. 2020 Nov 17;11:548904. doi: 10.3389/fpsyt.2020.548904 (PMC7718009; doi:10.3389/fpsyt.2020.548904)
Supplement: Supplementary file 2 [file Data_Sheet_1.docx]

**Supplementary Tables**

**Supplementary Table 1**. Medians and ranges of participant demographics and baseline clinical scores.

|  | ESC/MEM  Median (min-max) | ESC/PBO  Median (min-max) |
| --- | --- | --- |
|  | **N = 22** | **N = 16** |
| **Age** | **72.0 (60-82)** | **72.5 (60-83)** |
| **Education**  **in years** | **16 (12-20)** | **17 (13-18)** |
| **MMSE** | **28.5 (25-30)** | **28.5 (26-30)** |
| **HAMD** | **17 (16-26)** | **17 (16-23)** |
| **HAMA** | **10.5 (0-18)** | **9.5 (3-13)** |
| **AES** | **48.0 (31-63)** | **49 (29-65)** |

MMSE = Mini Mental State Examination; HAMD = Hamilton Depression Rating Scale; HAMA = Hamilton Anxiety Scale; AES = Apathy Evaluation Scale.

**Supplementary Table 2**. Medians and ranges of clinical scores at baseline and 6 months by treatment group for the 26 completers of the trial.

| **Variable** | **ESC/MEM**  **Median (min-max)**  **N=15** | | | **ESC/PBO**  **Median (min-max)**  **N=11** | | |
| --- | --- | --- | --- | --- | --- | --- |
|  | Baseline | 6 months | Change | Baseline | 6 months | Change |
| **HAMD** | 17  (16-26) | 4  (0-21) | -13  (-22 – 3) | 17  (16-22) | 5  (1-14) | -12  (-15 – 2) |
| **HAMA** | 10  (0-18) | 4  (0-11) | -5  (-14 – 1) | 11  (3-13) | 6  (3-12) | -4  (-7 -0) |
| **AES** | 48  (37-63) | 59  (46-65) | 9  (-1 -24) | 51.5 (40-65) | 62  (53-64) | 7  (-1-13) |

HAMD = Hamilton Depression Rating Scale; MMSE = Mini Mental State Examination; HAMA = Hamilton Anxiety Scale; AES = Apathy Evaluation Scale.

**Supplementary Table 3. Relationship between change in HAMD and baseline AD, MD and RD in ESC/MEM and ESC/PBO.** The results stem from the rank-based general linear models including the treatment group, AD/MD/RD and the interaction between group and AD/MD/RD as the predictors, while controlling for the respective baseline score, age, sex and scanner.

| **HAMD** | Left | | | | | | | | Right | | | |  |  |  |  |  |  |  |  |  |  |  |  |  |  |
| --- | --- | --- | --- | --- | --- | --- | --- | --- | --- | --- | --- | --- | --- | --- | --- | --- | --- | --- | --- | --- | --- | --- | --- | --- | --- | --- |
|  | **F(1,18)** | ***p*-value** | **Group** | **Beta (SE)** | | ***p*-value** | **Interaction group x AD/MD/RD** | | ***p*-value** | **Group** | **Beta (SE)** | ***p*-value** |  |  |  |  |  |  |  |  |  |  |  |  |  |  |
|  |  |  |  |  | |  |  | |  |  |  |  |  |  |  |  |  |  |  |  |  |  |  |  |  |  |
| **AD** |  |  |  |  | |  |  | |  |  |  |  |  |  |  |  |  |  |  |  |  |  |  |  |  |  |
| **ALIC** |  |  |  |  | |  |  | |  |  |  |  |  |  |  |  |  |  |  |  |  |  |  |  |  |  |
| AD | 0.61 | 0.44 |  |  | |  | 0.28 | | 0.61 |  |  |  |  |  |  |  |  |  |  |  |  |  |  |  |  |  |
| Group | 0.76 | 0.4 |  |  | |  | 2.18 | | 0.16 |  |  |  |  |  |  |  |  |  |  |  |  |  |  |  |  |  |
| Group x AD | 0.2 | 0.66 |  |  | |  | 1.22 | | 0.28 |  |  |  |  |  |  |  |  |  |  |  |  |  |  |  |  |  |
|  |  |  | ESC/MEM | 0.3 (0.36) | | 0.41 |  | |  | ESC/MEM | 0.42 (0.39) | 0.3 |  |  |  |  |  |  |  |  |  |  |  |  |  |  |
|  |  |  | ESC/PBO | 0.09 (0.33) | | 0.79 |  | |  | ESC/PBO | -0.1 (0.38) | 0.8 |  |  |  |  |  |  |  |  |  |  |  |  |  |  |
|  |  |  |  |  | |  |  | |  |  |  |  |  |  |  |  |  |  |  |  |  |  |  |  |  |  |
| **PLIC** |  |  |  |  | |  |  | |  |  |  |  |  |  |  |  |  |  |  |  |  |  |  |  |  |  |
| AD | 2.63 | 0.12 |  |  | |  | <0.01 | | 0.95 |  |  |  |  |  |  |  |  |  |  |  |  |  |  |  |  |  |
| Group | 0.23 | 0.63 |  |  | |  | 0.57 | | 0.46 |  |  |  |  |  |  |  |  |  |  |  |  |  |  |  |  |  |
| Group x AD | 1.42 | 0.25 |  |  | |  | 0.13 | | 0.72 |  |  |  |  |  |  |  |  |  |  |  |  |  |  |  |  |  |
|  |  |  | ESC/MEM | -0.65 (0.29) | | 0.04 |  | |  | ESC/MEM | 0.09 (0.32) | 0.79 |  |  |  |  |  |  |  |  |  |  |  |  |  |  |
|  |  |  | ESC/PBO | -0.07 (0.37) | | 0.86 |  | |  | ESC/PBO | -0.13 (0.51) | 0.81 |  |  |  |  |  |  |  |  |  |  |  |  |  |  |
|  |  |  |  |  | |  |  | |  |  |  |  |  |  |  |  |  |  |  |  |  |  |  |  |  |  |
| **CGC** |  |  |  |  | |  |  | |  |  |  |  |  |  |  |  |  |  |  |  |  |  |  |  |  |  |
| AD | 1.36 | 0.26 |  |  | |  | 0.98 | | 0.34 |  |  |  |  |  |  |  |  |  |  |  |  |  |  |  |  |  |
| Group | 0.91 | 0.35 |  |  | |  | 1.91 | | 0.18 |  |  |  |  |  |  |  |  |  |  |  |  |  |  |  |  |  |
| Group x AD | 0.75 | 0.4 |  |  | |  | 0.69 | | 0.42 |  |  |  |  |  |  |  |  |  |  |  |  |  |  |  |  |  |
|  |  |  | ESC/MEM | 0.51 (0.33) | | 0.15 |  | |  | ESC/MEM | -0.09 (0.36) | 0.8 |  |  |  |  |  |  |  |  |  |  |  |  |  |  |
|  |  |  | ESC/PBO | 0.1 (0.36) | | 0.78 |  | |  | ESC/PBO | -0.45 (0.34) | 0.2 |  |  |  |  |  |  |  |  |  |  |  |  |  |  |
|  |  |  |  |  | |  |  | |  |  |  |  |  |  |  |  |  |  |  |  |  |  |  |  |  |  |
| **IFO** |  |  |  |  | |  |  | |  |  |  |  |  |  |  |  |  |  |  |  |  |  |  |  |  |  |
| AD | 1.11 | 0.31 |  |  | |  | 1.82 | | 0.19 |  |  |  |  |  |  |  |  |  |  |  |  |  |  |  |  |  |
| Group | 0.35 | 0.56 |  |  | |  | 1.09 | | 0.31 |  |  |  |  |  |  |  |  |  |  |  |  |  |  |  |  |  |
| Group x AD | 1.97 | 0.18 |  |  | |  | 3.11 | | 0.09 |  |  |  |  |  |  |  |  |  |  |  |  |  |  |  |  |  |
|  |  |  | ESC/MEM | -0.55 (0.27) | | 0.06 |  | |  | ESC/MEM | -0.68 (0.3) | 0.04 |  |  |  |  |  |  |  |  |  |  |  |  |  |  |
|  |  |  | ESC/PBO | 0.07 (0.35) | | 0.85 |  | |  | ESC/PBO | 0.03 (0.33) | 0.92 |  |  |  |  |  |  |  |  |  |  |  |  |  |  |
|  |  |  |  |  | |  |  | |  |  |  |  |  |  |  |  |  |  |  |  |  |  |  |  |  |  |
| **SFO** |  |  |  |  | |  |  | |  |  |  |  |  |  |  |  |  |  |  |  |  |  |  |  |  |  |
| AD | 2.92 | 0.1 |  |  | |  | 0.22 | | 0.65 |  |  |  |  |  |  |  |  |  |  |  |  |  |  |  |  |  |
| Group | 0.01 | 0.92 |  |  | |  | <0.01 | | 0.95 |  |  |  |  |  |  |  |  |  |  |  |  |  |  |  |  |  |
| Group x AD | 0.35 | 0.56 |  |  | |  | 0.25 | | 0.63 |  |  |  |  |  |  |  |  |  |  |  |  |  |  |  |  |  |
|  |  |  | ESC/MEM | 0.31 (0.3) | | 0.33 |  | |  | ESC/MEM | 0.009 (0.3) | 0.98 |  |  |  |  |  |  |  |  |  |  |  |  |  |  |
|  |  |  | ESC/PBO | 0.56 (0.35) | | 0.13 |  | |  | ESC/PBO | 0.24 (0.4) | 0.56 |  |  |  |  |  |  |  |  |  |  |  |  |  |  |
|  |  |  |  |  | |  |  | |  |  |  |  |  |  |  |  |  |  |  |  |  |  |  |  |  |  |
| **SLF** |  |  |  |  | |  |  | |  |  |  |  |  |  |  |  |  |  |  |  |  |  |  |  |  |  |
| AD | 6.86 | 0.02 |  |  | |  | 0.75 | | 0.4 |  |  |  |  |  |  |  |  |  |  |  |  |  |  |  |  |  |
| Group | 0.71 | 0.41 |  |  | |  | 1.84 | | 0.19 |  |  |  |  |  |  |  |  |  |  |  |  |  |  |  |  |  |
| Group x AD | 0.63 | 0.44 |  |  | |  | 1.18 | | 0.29 |  |  |  |  |  |  |  |  |  |  |  |  |  |  |  |  |  |
|  |  |  | ESC/MEM | 0.78 (0.26) | | 0.007 |  | |  | ESC/MEM | 0.49 (0.29) | 0.11 |  |  |  |  |  |  |  |  |  |  |  |  |  |  |
|  |  |  | ESC/PBO | 0.45 (0.36) | | 0.23 |  | |  | ESC/PBO | -0.02 (0.42) | 0.97 |  |  |  |  |  |  |  |  |  |  |  |  |  |  |
|  |  |  |  |  | |  |  | |  |  |  |  |  |  |  |  |  |  |  |  |  |  |  |  |  |  |
| **FX** |  |  |  |  | |  |  | |  |  |  |  |  |  |  |  |  |  |  |  |  |  |  |  |  |  |
| AD | 0.29 | 0.6 |  |  | |  | <0.01 | | 0.95 |  |  |  |  |  |  |  |  |  |  |  |  |  |  |  |  |  |
| Group | 1.06 | 0.32 |  |  | |  | 1.62 | | 0.22 |  |  |  |  |  |  |  |  |  |  |  |  |  |  |  |  |  |
| Group x AD | 0.58 | 0.46 |  |  | |  | 0.8 | | 0.38 |  |  |  |  |  |  |  |  |  |  |  |  |  |  |  |  |  |
|  |  |  | ESC/MEM | 0.36 (0.34) | | 0.3 |  | |  | ESC/MEM | 0.25 (0.35) | 0.49 |  |  |  |  |  |  |  |  |  |  |  |  |  |  |
|  |  |  | ESC/PBO | -0.07 (0.44) | | 0.87 |  | |  | ESC/PBO | -0.21 (0.42) | 0.62 |  |  |  |  |  |  |  |  |  |  |  |  |  |  |
|  |  |  |  |  | |  |  | |  |  |  |  |  |  |  |  |  |  |  |  |  |  |  |  |  |  |
|  | **F(1,18)** | ***p*-value** | **Group** | | **Beta (SE)** | | | ***p*-value** | | | | |  |  |  |  |  |  |  |  |  |  |  |  |  |  |
| **FX Body** |  |  |  | |  | | |  | | | | |  |  |  |  |  |  |  |  |  |  |  |  |  |  |
| AD | 2.03 | 0.17 |  | |  | | |  | | | | |  |  |  |  |  |  |  |  |  |  |  |  |  |  |
| Group | 0.06 | 0.82 |  | |  | | |  | | | | |  |  |  |  |  |  |  |  |  |  |  |  |  |  |
| Group x AD | 0.01 | 0.91 |  | |  | | |  | | | | |  |  |  |  |  |  |  |  |  |  |  |  |  |  |
|  |  |  | ESC/MEM | | 0.32 (0.31) | | | 0.31 | | | | |  |  | | | | | | ESC/MEM | | | | | |  |
|  |  |  | ESC/PBO | | 0.37 (0.34) | | | 0.29 | | | | |  |  | | | | | | ESC/PBO | | | | | |  |
|  |  |  |  | |  | | |  | | | | |  |  |  |  |  |  |  |  |  |  |  |  |  |  |
| **GCC** |  |  |  | |  | | |  | | | | |  |  |  |  |  |  |  |  |  |  |  |  |  |  |
| AD | 0.19 | 0.67 |  | |  | | |  | | | | |  |  |  |  |  |  |  |  |  |  |  |  |  |  |
| Group | 0.59 | 0.45 |  | |  | | |  | | | | |  |  |  |  |  |  |  |  |  |  |  |  |  |  |
| Group x AD | 0.31 | 0.58 |  | |  | | |  | | | | |  |  |  |  |  |  |  |  |  |  |  |  |  |  |
|  |  |  | ESC/MEM | | 0.33 (0.35) | | | 0.36 | | | | |  | |  | | | | | | ESC/MEM | | | | | |
|  |  |  | ESC/PBO | | 0.03 (0.61) | | | 0.96 | | | | |  | |  | | | | | | ESC/PBO | | | | | |
|  |  |  |  | |  | | |  | | | | |  |  |  |  |  |  |  |  |  |  |  |  |  |  |
|  |  |  |  |  | |  |  | |  |  |  |  |  |  |  |  |  |  |  |  |  |  |  |  |  |  |
| **MD** |  |  |  |  | |  |  | |  |  |  |  |  |  |  |  |  |  |  |  |  |  |  |  |  |  |
| **ALIC** |  |  |  |  | |  |  | |  |  |  |  |  |  |  |  |  |  |  |  |  |  |  |  |  |  |
| MD | 4.64 | 0.04 |  |  | |  | 0.88 | | 0.36 |  |  |  |  |  |  |  |  |  |  |  |  |  |  |  |  |  |
| Group | 4.63 | 0.05 |  |  | |  | 5.11 | | .04 |  |  |  |  |  |  |  |  |  |  |  |  |  |  |  |  |  |
| Group x MD | 3.91 | 0.06 |  |  | |  | 4.28 | | 0.05 |  |  |  |  |  |  |  |  |  |  |  |  |  |  |  |  |  |
|  |  |  | ESC/MEM | 0.82 (0.26) | | 0.005 |  | |  | ESC/MEM | 0.72 (0.32) | 0.04 |  |  |  |  |  |  |  |  |  |  |  |  |  |  |
|  |  |  | ESC/PBO | 0.1 (0.3) | | 0.73 |  | |  | ESC/PBO | -0.11 (0.43) | 0.79 |  |  |  |  |  |  |  |  |  |  |  |  |  |  |
|  |  |  |  |  | |  |  | |  |  |  |  |  |  |  |  |  |  |  |  |  |  |  |  |  |  |
| **PLIC** |  |  |  |  | |  |  | |  |  |  |  |  |  |  |  |  |  |  |  |  |  |  |  |  |  |
| MD | 0.23 | 0.64 |  |  | |  | <0.01 | | 0.99 |  |  |  |  |  |  |  |  |  |  |  |  |  |  |  |  |  |
| Group | 5.45 | 0.03 |  |  | |  | 5.17 | | 0.04 |  |  |  |  |  |  |  |  |  |  |  |  |  |  |  |  |  |
| Group x MD | 4.12 | 0.06 |  |  | |  | 3.92 | | 0.06 |  |  |  |  |  |  |  |  |  |  |  |  |  |  |  |  |  |
|  |  |  | ESC/MEM | 0.3 (0.3) | | 0.33 |  | |  | ESC/MEM | 0.4 (0.33) | 0.24 |  |  |  |  |  |  |  |  |  |  |  |  |  |  |
|  |  |  | ESC/PBO | -0.58 (0.41) | | 0.18 |  | |  | ESC/PBO | -0.42 (0.41) | 0.32 |  |  |  |  |  |  |  |  |  |  |  |  |  |  |
|  |  |  |  |  | |  |  | |  |  |  |  |  |  |  |  |  |  |  |  |  |  |  |  |  |  |
| **CGC** |  |  |  |  | |  |  | |  |  |  |  |  |  |  |  |  |  |  |  |  |  |  |  |  |  |
| MD | 0.79 | 0.39 |  |  | |  | 1.76 | | 0.2 |  |  |  |  |  |  |  |  |  |  |  |  |  |  |  |  |  |
| Group | 2.96 | 0.1 |  |  | |  | 1.8 | | 0.2 |  |  |  |  |  |  |  |  |  |  |  |  |  |  |  |  |  |
| Group x MD | 1.53 | 0.23 |  |  | |  | 1.27 | | 0.27 |  |  |  |  |  |  |  |  |  |  |  |  |  |  |  |  |  |
|  |  |  | ESC/MEM | 0.52 (0.32) | | 0.12 |  | |  | ESC/MEM | 0.64 (0.35) | 0.09 |  |  |  |  |  |  |  |  |  |  |  |  |  |  |
|  |  |  | ESC/PBO | 0.01 (0.41) | | 0.98 |  | |  | ESC/PBO | 0.16 (0.38) | 0.69 |  |  |  |  |  |  |  |  |  |  |  |  |  |  |
|  |  |  |  |  | |  |  | |  |  |  |  |  |  |  |  |  |  |  |  |  |  |  |  |  |  |
| **IFO** |  |  |  |  | |  |  | |  |  |  |  |  |  |  |  |  |  |  |  |  |  |  |  |  |  |
| MD | 0.26 | 0.62 |  |  | |  | 0.66 | | 0.43 |  |  |  |  |  |  |  |  |  |  |  |  |  |  |  |  |  |
| Group | 6.34 | 0.02 |  |  | |  | 1.99 | | 0.18 |  |  |  |  |  |  |  |  |  |  |  |  |  |  |  |  |  |
| Group x MD | 5.36 | 0.03 |  |  | |  | 1.06 | | 0.32 |  |  |  |  |  |  |  |  |  |  |  |  |  |  |  |  |  |
|  |  |  | ESC/MEM | 0.57 (0.26) | | 0.04 |  | |  | ESC/MEM | 0.42 (0.28) | 0.16 |  |  |  |  |  |  |  |  |  |  |  |  |  |  |
|  |  |  | ESC/PBO | -0.37 (0.31) | | 0.26 |  | |  | ESC/PBO | -0.05 (0.35) | 0.9 |  |  |  |  |  |  |  |  |  |  |  |  |  |  |
|  |  |  |  |  | |  |  | |  |  |  |  |  |  |  |  |  |  |  |  |  |  |  |  |  |  |
| **SFO** |  |  |  |  | |  |  | |  |  |  |  |  |  |  |  |  |  |  |  |  |  |  |  |  |  |
| MD | 0.76 | 0.39 |  |  | |  | 1.84 | | 0.19 |  |  |  |  |  |  |  |  |  |  |  |  |  |  |  |  |  |
| Group | 0.84 | 0.37 |  |  | |  | 3.63 | | 0.07 |  |  |  |  |  |  |  |  |  |  |  |  |  |  |  |  |  |
| Group x MD | 0.66 | 0.43 |  |  | |  | 3.74 | | 0.07 |  |  |  |  |  |  |  |  |  |  |  |  |  |  |  |  |  |
|  |  |  | ESC/MEM | 0.45 (0.29) | | 0.14 |  | |  | ESC/MEM | 0.75 (0.3) | 0.02 |  |  |  |  |  |  |  |  |  |  |  |  |  |  |
|  |  |  | ESC/PBO | 0.02 (0.45) | | 0.96 |  | |  | ESC/PBO | -0.1 (0.34) | 0.78 |  |  |  |  |  |  |  |  |  |  |  |  |  |  |
|  |  |  |  |  | |  |  | |  |  |  |  |  |  |  |  |  |  |  |  |  |  |  |  |  |  |
| **SLF** |  |  |  |  | |  |  | |  |  |  |  |  |  |  |  |  |  |  |  |  |  |  |  |  |  |
| MD | 4.22 | 0.05 |  |  | |  | 4.26 | | 0.05 |  |  |  |  |  |  |  |  |  |  |  |  |  |  |  |  |  |
| Group | 2.89 | 0.11 |  |  | |  | 2.89 | | 0.11 |  |  |  |  |  |  |  |  |  |  |  |  |  |  |  |  |  |
| Group x MD | 2.39 | 0.14 |  |  | |  | 3.18 | | 0.09 |  |  |  |  |  |  |  |  |  |  |  |  |  |  |  |  |  |
|  |  |  | ESC/MEM | 0.7 (0.27) | | 0.02 |  | |  | ESC/MEM | 0.83 (0.26) | 0.005 |  |  |  |  |  |  |  |  |  |  |  |  |  |  |
|  |  |  | ESC/PBO | 0.1 (0.28) | | 0.74 |  | |  | ESC/PBO | 0.07 (0.35) | 0.84 |  |  |  |  |  |  |  |  |  |  |  |  |  |  |
|  |  |  |  |  | |  |  | |  |  |  |  |  |  |  |  |  |  |  |  |  |  |  |  |  |  |
| **FX** |  |  |  |  | |  |  | |  |  |  |  |  |  |  |  |  |  |  |  |  |  |  |  |  |  |
| MD | 1.0 | 0.33 |  |  | |  | 0.37 | | 0.55 |  |  |  |  |  |  |  |  |  |  |  |  |  |  |  |  |  |
| Group | 0.51 | 0.48 |  |  | |  | 1.77 | | 0.2 |  |  |  |  |  |  |  |  |  |  |  |  |  |  |  |  |  |
| Group x MD | 0.11 | 0.75 |  |  | |  | 1.07 | | 0.31 |  |  |  |  |  |  |  |  |  |  |  |  |  |  |  |  |  |
|  |  |  | ESC/MEM | 0.37 (0.33) | | 0.28 |  | |  | ESC/MEM | 0.44 (0.34) | 0.21 |  |  |  |  |  |  |  |  |  |  |  |  |  |  |
|  |  |  | ESC/PBO | 0.22 (0.41) | | 0.6 |  | |  | ESC/PBO | -0.06 (0.45) | 0.9 |  |  |  |  |  |  |  |  |  |  |  |  |  |  |
|  |  |  |  |  | |  |  | |  |  |  |  |  |  |  |  |  |  |  |  |  |  |  |  |  |  |
| **FX Body** | **F(1,18)** | ***p*-value** | **Group** | | **Beta (SE)** | | | ***p*-value** | | | | |  |  |  |  |  |  |  |  |  |  |  |  |  |  |
| MD | 2.21 | 0.15 |  | |  | | |  | | | | |  |  |  |  |  |  |  |  |  |  |  |  |  |  |
| Group | 0.31 | 0.59 |  | |  | | |  | | | | |  |  |  |  |  |  |  |  |  |  |  |  |  |  |
| Group x MD | 0.01 | 0.93 |  | |  | | |  | | | | |  |  |  |  |  |  |  |  |  |  |  |  |  |  |
|  |  |  | ESC/MEM | | 0.39 (0.31) | | | 0.23 | | | | |  | | |  | | | | | | ESC/MEM | | | | |
|  |  |  | ESC/PBO | | 0.35 (0.34) | | | 0.31 | | | | |  | | |  | | | | | | ESC/PBO | | | | |
|  |  |  |  | |  | | |  | | | | |  |  |  |  |  |  |  |  |  |  |  |  |  |  |
| **GCC** |  |  |  | |  | | |  | | | | |  |  |  |  |  |  |  |  |  |  |  |  |  |  |
| MD | 2.07 | 0.17 |  | |  | | |  | | | | |  |  |  |  |  |  |  |  |  |  |  |  |  |  |
| Group | 0.53 | 0.48 |  | |  | | |  | | | | |  |  |  |  |  |  |  |  |  |  |  |  |  |  |
| Group x MD | 0.21 | 0.65 |  | |  | | |  | | | | |  |  |  |  |  |  |  |  |  |  |  |  |  |  |
|  |  |  | ESC/MEM | | 0.49 (0.3) | | | 0.12 | | | | |  | | | |  | | | | | | ESC/MEM | | | |
|  |  |  | ESC/PBO | | 0.28 (0.4) | | | 0.5 | | | | |  | | | |  | | | | | | ESC/PBO | | | |
|  |  |  |  | |  | | |  | | | | |  |  |  |  |  |  |  |  |  |  |  |  |  |  |
|  |  |  |  |  | |  |  | |  |  |  |  |  |  |  |  |  |  |  |  |  |  |  |  |  |  |
| **RD** |  |  |  |  | |  |  | |  |  |  |  |  |  |  |  |  |  |  |  |  |  |  |  |  |  |
| **ALIC** |  |  |  |  | |  |  | |  |  |  |  |  |  |  |  |  |  |  |  |  |  |  |  |  |  |
| RD | 5.37 | 0.03 |  |  | |  | 3.63 | | 0.07 |  |  |  |  |  |  |  |  |  |  |  |  |  |  |  |  |  |
| Group | 5.56 | 0.03 |  |  | |  | 5.62 | | 0.03 |  |  |  |  |  |  |  |  |  |  |  |  |  |  |  |  |  |
| Group x RD | 3.97 | 0.06 |  |  | |  | 5.07 | | 0.04 |  |  |  |  |  |  |  |  |  |  |  |  |  |  |  |  |  |
|  |  |  | ESC/MEM | 0.83 (0.27) | | 0.007 |  | |  | ESC/MEM | 0.96 (0.29) | 0.004 |  |  |  |  |  |  |  |  |  |  |  |  |  |  |
|  |  |  | ESC/PBO | 0.12 (0.27) | | 0.66 |  | |  | ESC/PBO | 0.12 (0.38) | 0.76 |  |  |  |  |  |  |  |  |  |  |  |  |  |  |
|  |  |  |  |  | |  |  | |  |  |  |  |  |  |  |  |  |  |  |  |  |  |  |  |  |  |
| **PLIC** |  |  |  |  | |  |  | |  |  |  |  |  |  |  |  |  |  |  |  |  |  |  |  |  |  |
| RD | 0.02 | 0.88 |  |  | |  | 0.01 | | 0.94 |  |  |  |  |  |  |  |  |  |  |  |  |  |  |  |  |  |
| Group | 4.92 | 0.04 |  |  | |  | 5.47 | | 0.03 |  |  |  |  |  |  |  |  |  |  |  |  |  |  |  |  |  |
| Group x RD | 4.09 | 0.06 |  |  | |  | 4.31 | | 0.05 |  |  |  |  |  |  |  |  |  |  |  |  |  |  |  |  |  |
|  |  |  | ESC/MEM | 0.49 (0.3) | | 0.11 |  | |  | ESC/MEM | 0.43 (0.32) | 0.21 |  |  |  |  |  |  |  |  |  |  |  |  |  |  |
|  |  |  | ESC/PBO | -0.41 (0.39) | | 0.31 |  | |  | ESC/PBO | -0.47 (0.42) | 0.28 |  |  |  |  |  |  |  |  |  |  |  |  |  |  |
|  |  |  |  |  | |  |  | |  |  |  |  |  |  |  |  |  |  |  |  |  |  |  |  |  |  |
| **CGC** |  |  |  |  | |  |  | |  |  |  |  |  |  |  |  |  |  |  |  |  |  |  |  |  |  |
| RD | 1.62 | 0.22 |  |  | |  | 3.22 | | 0.09 |  |  |  |  |  |  |  |  |  |  |  |  |  |  |  |  |  |
| Group | 2.99 | 0.1 |  |  | |  | 2.58 | | 0.13 |  |  |  |  |  |  |  |  |  |  |  |  |  |  |  |  |  |
| Group x RD | 1.76 | 0.21 |  |  | |  | 2.44 | | 0.14 |  |  |  |  |  |  |  |  |  |  |  |  |  |  |  |  |  |
|  |  |  | ESC/MEM | 0.59 (0.26) | | 0.03 |  | |  | ESC/MEM | 0.78 (0.33) | 0.03 |  |  |  |  |  |  |  |  |  |  |  |  |  |  |
|  |  |  | ESC/PBO | 0.06 (0.39) | | 0.88 |  | |  | ESC/PBO | 0.14 (0.33) | 0.68 |  |  |  |  |  |  |  |  |  |  |  |  |  |  |
|  |  |  |  |  | |  |  | |  |  |  |  |  |  |  |  |  |  |  |  |  |  |  |  |  |  |
| **IFO** |  |  |  |  | |  |  | |  |  |  |  |  |  |  |  |  |  |  |  |  |  |  |  |  |  |
| RD | 0.81 | 0.38 |  |  | |  | 1.04 | | 0.32 |  |  |  |  |  |  |  |  |  |  |  |  |  |  |  |  |  |
| Group | 4.16 | 0.06 |  |  | |  | 1.91 | | 0.18 |  |  |  |  |  |  |  |  |  |  |  |  |  |  |  |  |  |
| Group x RD | 3.19 | 0.09 |  |  | |  | 1.04 | | 0.32 |  |  |  |  |  |  |  |  |  |  |  |  |  |  |  |  |  |
|  |  |  | ESC/MEM | 0.62 (0.33) | | 0.08 |  | |  | ESC/MEM | 0.46 (0.29) | 0.14 |  |  |  |  |  |  |  |  |  |  |  |  |  |  |
|  |  |  | ESC/PBO | -0.21 (0.32) | | 0.52 |  | |  | ESC/PBO | -0.003 (0.34) | 0.99 |  |  |  |  |  |  |  |  |  |  |  |  |  |  |
|  |  |  |  |  | |  |  | |  |  |  |  |  |  |  |  |  |  |  |  |  |  |  |  |  |  |
| **SFO** |  |  |  |  | |  |  | |  |  |  |  |  |  |  |  |  |  |  |  |  |  |  |  |  |  |
| RD | 2.04 | 0.17 |  |  | |  | 2.05 | | 0.17 |  |  |  |  |  |  |  |  |  |  |  |  |  |  |  |  |  |
| Group | 0.07 | 0.79 |  |  | |  | 1.4 | | 0.25 |  |  |  |  |  |  |  |  |  |  |  |  |  |  |  |  |  |
| Group x RD | <.01 | 0.96 |  |  | |  | 1.11 | | 0.31 |  |  |  |  |  |  |  |  |  |  |  |  |  |  |  |  |  |
|  |  |  | ESC/MEM | 0.35 (0.29) | | 0.25 |  | |  | ESC/MEM | 0.56 (0.28) | 0.06 |  |  |  |  |  |  |  |  |  |  |  |  |  |  |
|  |  |  | ESC/PBO | 0.32 (0.37) | | 0.39 |  | |  | ESC/PBO | 0.1 (0.35) | 0.78 |  |  |  |  |  |  |  |  |  |  |  |  |  |  |
|  |  |  |  |  | |  |  | |  |  |  |  |  |  |  |  |  |  |  |  |  |  |  |  |  |  |
| **SLF** |  |  |  |  | |  |  | |  |  |  |  |  |  |  |  |  |  |  |  |  |  |  |  |  |  |
| RD | 5.47 | 0.03 |  |  | |  | 5.63 | | 0.03 |  |  |  |  |  |  |  |  |  |  |  |  |  |  |  |  |  |
| Group | 3.25 | 0.09 |  |  | |  | 2.96 | | 0.1 |  |  |  |  |  |  |  |  |  |  |  |  |  |  |  |  |  |
| Group x RD | 2.67 | 0.12 |  |  | |  | 3.65 | | 0.07 |  |  |  |  |  |  |  |  |  |  |  |  |  |  |  |  |  |
|  |  |  | ESC/MEM | 0.74 (0.24) | | 0.006 |  | |  | ESC/MEM | 0.85 (0.23) | 0.001 |  |  |  |  |  |  |  |  |  |  |  |  |  |  |
|  |  |  | ESC/PBO | 0.13 (0.28) | | 0.65 |  | |  | ESC/PBO | 0.1 (0.33) | 0.77 |  |  |  |  |  |  |  |  |  |  |  |  |  |  |
|  |  |  |  |  | |  |  | |  |  |  |  |  |  |  |  |  |  |  |  |  |  |  |  |  |  |
| **FX** |  |  |  |  | |  |  | |  |  |  |  |  |  |  |  |  |  |  |  |  |  |  |  |  |  |
| RD | 1.17 | 0.29 |  |  | |  | 0.69 | | 0.42 |  |  |  |  |  |  |  |  |  |  |  |  |  |  |  |  |  |
| Group | 0.86 | 0.37 |  |  | |  | 1.3 | | 0.27 |  |  |  |  |  |  |  |  |  |  |  |  |  |  |  |  |  |
| Group x RD | 0.4 | 0.53 |  |  | |  | 0.66 | | 0.43 |  |  |  |  |  |  |  |  |  |  |  |  |  |  |  |  |  |
|  |  |  | ESC/MEM | 0.45 (0.34) | | 0.21 |  | |  | ESC/MEM | 0.47 (0.34) | 0.18 |  |  |  |  |  |  |  |  |  |  |  |  |  |  |
|  |  |  | ESC/PBO | 0.17 (0.38) | | 0.67 |  | |  | ESC/PBO | 0.07 (0.47) | 0.88 |  |  |  |  |  |  |  |  |  |  |  |  |  |  |
|  |  |  |  |  | |  |  | |  |  |  |  |  |  |  |  |  |  |  |  |  |  |  |  |  |  |
|  | **F(1,18)** | ***p*-value** | **Group** | | **Beta (SE)** | | | ***p*-value** | | | | |  |  |  |  |  |  |  |  |  |  |  |  |  |  |
| **FX Body** |  |  |  | |  | | |  | | | | |  |  |  |  |  |  |  |  |  |  |  |  |  |  |
| RD | 2.22 | 0.15 |  | |  | | |  | | | | |  |  |  |  |  |  |  |  |  |  |  |  |  |  |
| Group | 0.33 | 0.58 |  | |  | | |  | | | | |  |  |  |  |  |  |  |  |  |  |  |  |  |  |
| Group x RD | 0.02 | 0.89 |  | |  | | |  | | | | |  |  |  |  |  |  |  |  |  |  |  |  |  |  |
|  |  |  | ESC/MEM | | 0.4 (0.31) | | | 0.21 | | | | |  | | | | |  | | | | | | ESC/MEM | | |
|  |  |  | ESC/PBO | | 0.34 (0.34) | | | 0.33 | | | | |  | | | | |  | | | | | | ESC/PBO | | |
|  |  |  |  | |  | | |  | | | | |  |  |  |  |  |  |  |  |  |  |  |  |  |  |
| **GCC** |  |  |  | |  | | |  | | | | |  |  |  |  |  |  |  |  |  |  |  |  |  |  |
| RD | 2.26 | 0.15 |  | |  | | |  | | | | |  |  |  |  |  |  |  |  |  |  |  |  |  |  |
| Group | 0.52 | 0.48 |  | |  | | |  | | | | |  |  |  |  |  |  |  |  |  |  |  |  |  |  |
| Group x RD | 0.15 | 0.71 |  | |  | | |  | | | | |  |  |  |  |  |  |  |  |  |  |  |  |  |  |
|  |  |  | ESC/MEM | | 0.47 (0.3) | | | 0.13 | | | | |  | | | | | |  | | | | | | ESC/MEM | |
|  |  |  | ESC/PBO | | 0.3 (0.38) | | | 0.44 | | | | |  | | | | | |  | | | | | | ESC/PBO | |
|  |  |  |  | |  | | |  | | | | |  |  |  |  |  |  |  |  |  |  |  |  |  |  |

HAMD = Hamilton Depression Scale.

**Supplementary Table 4. Associations between baseline FA and change in related clinical scores.** The results stem from the rank-based general linear models including the treatment group, FA and the interaction between group and FA as the predictor, while controlling for the respective baseline score, age, sex and scanner. Higher baseline FA was associated with larger HAMA score improvements in the left ALIC and SLF, as well as in bilateral CGC and IFO only in the ESC+MEM group. For apathy, higher FA in the right SFO was associated with better treatment outcome in the ESC+MEM group.

| **FA** | **Left** | | | | | **Right** | | | | |
| --- | --- | --- | --- | --- | --- | --- | --- | --- | --- | --- |
|  | **F(1,18)** | ***p*-value** | **Group** | **Beta (SE)** | ***p*-value** | **F(1,18)** | ***p*-value** | **Group** | **Beta (SE)** | ***p*-value** |
|  |  |  |  |  |  |  |  |  |  |  |
| **HAMA** |  |  |  |  |  |  |  |  |  |  |
| **ALIC** |  |  |  |  |  |  |  |  |  |  |
| FA | 3.79 | 0.07 |  |  |  | 4.2 | 0.06 |  |  |  |
| Group | 1.12 | 0.3 |  |  |  | 0.06 | 0.82 |  |  |  |
| Group x FA | 4.95 | 0.04 |  |  |  | 0.65 | 0.43 |  |  |  |
|  |  |  | ESC/MEM | -0.65 (0.19) | 0.004 |  |  | ESC/MEM | -0.61 (0.23) | 0.02 |
|  |  |  | ESC/PBO | 0.06 (0.24) | 0.8 |  |  | ESC/PBO | -0.31 (0.34) | 0.38 |
|  |  |  |  |  |  |  |  |  |  |  |
| **PLIC** |  |  |  |  |  |  |  |  |  |  |
| FA | <0.01 | 0.97 |  |  |  | 1.07 | 0.32 |  |  |  |
| Group | 1.13 | 0.3 |  |  |  | 0.42 | 0.53 |  |  |  |
| Group x FA | 3.54 | 0.08 |  |  |  | 1.59 | 0.22 |  |  |  |
|  |  |  | ESC/MEM | -0.4 (0.22) | 0.08 |  |  | ESC/MEM | -0.54 (0.3) | 0.09 |
|  |  |  | ESC/PBO | 0.38 (0.35) | 0.28 |  |  | ESC/PBO | 0.03 (0.37) | 0.93 |
|  |  |  |  |  |  |  |  |  |  |  |
| **CGC** |  |  |  |  |  |  |  |  |  |  |
| FA | 0.2 | 0.66 |  |  |  | 0.88 | 0.36 |  |  |  |
| Group | 2.56 | 0.13 |  |  |  | 1.43 | 0.25 |  |  |  |
| Group x FA | 8.3 | 0.01 |  |  |  | 4.89 | 0.04 |  |  |  |
|  |  |  | ESC/MEM | -0.56 (0.2) | 0.01 |  |  | ESC/MEM | -0.57 (0.23) | 0.03 |
|  |  |  | ESC/PBO | 0.39 (0.29) | 0.19 |  |  | ESC/PBO | 0.22 (0.28) | 0.43 |
|  |  |  |  |  |  |  |  |  |  |  |
| **IFO** |  |  |  |  |  |  |  |  |  |  |
| FA | 1.31 | 0.27 |  |  |  | 0.92 | 0.35 |  |  |  |
| Group | 1.78 | 0.2 |  |  |  | 2.42 | 0.14 |  |  |  |
| Group x FA | 6.02 | 0.02 |  |  |  | 6.17 | 0.02 |  |  |  |
|  |  |  | ESC/MEM | -0.6 (0.24) | 0.02 |  |  | ESC/MEM | -0.6 (0.2) | 0.007 |
|  |  |  | ESC/PBO | 0.22 (0.23) | 0.34 |  |  | ESC/PBO | 0.25 (0.29) | 0.41 |
|  |  |  |  |  |  |  |  |  |  |  |
| **SFO** |  |  |  |  |  |  |  |  |  |  |
| FA | 0.62 | 0.44 |  |  |  | 13.10 | 0.002 |  |  |  |
| Group | 0.06 | 0.81 |  |  |  | 1.63 | 0.22 |  |  |  |
| Group x FA | 0.67 | 0.43 |  |  |  | 3.13 | 0.09 |  |  |  |
|  |  |  | ESC/MEM | -0.34 (0.25) | 0.2 |  |  | ESC/MEM | -0.88 (0.25) | 0.002 |
|  |  |  | ESC/PBO | 0.01 (0.33) | 0.97 |  |  | ESC/PBO | -0.32 (0.21) | 0.14 |
|  |  |  |  |  |  |  |  |  |  |  |
| **SLF** |  |  |  |  |  |  |  |  |  |  |
| FA | 2.82 | 0.11 |  |  |  | 6.95 | 0.02 |  |  |  |
| Group | 1.06 | 0.32 |  |  |  | 0.12 | 0.73 |  |  |  |
| Group x FA | 4.34 | 0.05 |  |  |  | 0.45 | 0.51 |  |  |  |
|  |  |  | ESC/MEM | -0.62 (0.24) | 0.02 |  |  | ESC/MEM | -0.58 (0.21) | 0.01 |
|  |  |  | ESC/PBO | 0.07 (0.22) | 0.76 |  |  | ESC/PBO | -0.34 (0.28) | 0.23 |
|  |  |  |  |  |  |  |  |  |  |  |
| **FX** |  |  |  |  |  |  |  |  |  |  |
| FA | 0.16 | 0.7 |  |  |  | 0.11 | 0.74 |  |  |  |
| Group | 0.42 | 0.53 |  |  |  | 0.12 | 0.73 |  |  |  |
| Group x FA | <0.01 | 1.0 |  |  |  | 1.16 | 0.3 |  |  |  |
|  |  |  | ESC/MEM | -0.11 (0.3) | 0.71 |  |  | ESC/MEM | -0.33 (0.28) | 0.25 |
|  |  |  | ESC/PBO | -0.11 (0.39) | 0.78 |  |  | ESC/PBO | 0.13 (0.43) | 0.76 |
|  |  |  |  |  |  |  |  |  |  |  |
|  | **F(1,18)** | ***p*-value** | **Group** | **Beta (SE)** | ***p*-value** | | | | | |
| **FX Body** |  |  |  |  |  |  |  |  |  |  |
| FA | 1.29 | 0.27 |  |  |  |  |  |  |  |  |
| Group | 0.05 | 0.83 |  |  |  |  |  |  |  |  |
| Group x FA | 1.3 | 0.27 |  |  |  |  |  |  |  |  |
|  |  |  | ESC/MEM | -0.45 (0.26) | 0.1 | | | | | |
|  |  |  | ESC/PBO | -0.04 (0.3) | 0.9 | | | | | |
|  |  |  |  |  |  |  |  |  |  |  |
| **GCC** |  |  |  |  |  |  |  |  |  |  |
| FA | 0.23 | 0.64 |  |  |  |  |  |  |  |  |
| Group | 0.13 | 0.72 |  |  |  |  |  |  |  |  |
| Group x FA | 1.29 | 0.27 |  |  |  |  |  |  |  |  |
|  |  |  | ESC/MEM | -0.33 (0.24) | 0.18 | | | | | |
|  |  |  | ESC/PBO | 0.14 (0.34) | 0.69 | | | | | |
|  |  |  |  |  |  |  |  |  |  |  |
|  |  |  |  |  |  |  |  |  |  |  |
| **AES** |  |  |  |  |  |  |  |  |  |  |
| **ALIC** |  |  |  |  |  |  |  |  |  |  |
| FA | 8.93 | 0.008 |  |  |  | 1.9 | 0.19 |  |  |  |
| Group | 0.36 | 0.56 |  |  |  | 1.79 | 0.2 |  |  |  |
| Group x FA | 0.72 | 0.41 |  |  |  | 1.96 | 0.18 |  |  |  |
|  |  |  | ESC/MEM | 0.49 (0.15) | 0.005 |  |  | ESC/MEM | 0.48 (0.19) | 0.02 |
|  |  |  | ESC/PBO | 0.27 (0.21) | 0.22 |  |  | ESC/PBO | 0.05 (0.29) | 0.86 |
|  |  |  |  |  |  |  |  |  |  |  |
| **PLIC** |  |  |  |  |  |  |  |  |  |  |
| FA | 18.17 | 0.0005 |  |  |  | 5.52 | 0.03 |  |  |  |
| Group | 0.47 | 0.5 |  |  |  | 0.08 | 0.79 |  |  |  |
| Group x FA | 0.52 | 0.48 |  |  |  | 0.1 | 0.76 |  |  |  |
|  |  |  | ESC/MEM | 0.44 (0.13) | 0.004 |  |  | ESC/MEM | 0.49 (0.2) | 0.03 |
|  |  |  | ESC/PBO | 0.62 (0.21) | 0.009 |  |  | ESC/PBO | 0.4 (0.27) | 0.16 |
|  |  |  |  |  |  |  |  |  |  |  |
| **CGC** |  |  |  |  |  |  |  |  |  |  |
| FA | 0.2 | 0.66 |  |  |  | 0.96 | 0.34 |  |  |  |
| Group | 0.58 | 0.46 |  |  |  | 0.13 | 0.72 |  |  |  |
| Group x FA | 1.21 | 0.29 |  |  |  | 0.27 | 0.61 |  |  |  |
|  |  |  | ESC/MEM | 0.26 (0.2) | 0.21 |  |  | ESC/MEM | 0.26 (0.22) | 0.26 |
|  |  |  | ESC/PBO | -0.09 (0.3) | 0.77 |  |  | ESC/PBO | 0.09 (0.26) | 0.73 |
|  |  |  |  |  |  |  |  |  |  |  |
| **IFO** |  |  |  |  |  |  |  |  |  |  |
| FA | 7.2 | 0.02 |  |  |  | 1.09 | 0.31 |  |  |  |
| Group | 0.09 | 0.77 |  |  |  | 2.84 | 0.11 |  |  |  |
| Group x FA | 0.21 | 0.66 |  |  |  | 3.46 | 0.08 |  |  |  |
|  |  |  | ESC/MEM | 0.43 (0.19) | 0.04 |  |  | ESC/MEM | 0.45 (0.17) | 0.02 |
|  |  |  | ESC/PBO | 0.3 (0.19) | 0.14 |  |  | ESC/PBO | -0.12 (0.26) | 0.65 |
|  |  |  |  |  |  |  |  |  |  |  |
| **SFO** |  |  |  |  |  |  |  |  |  |  |
| FA | 4.29 | 0.05 |  |  |  | 4.55 | 0.05 |  |  |  |
| Group | 0.89 | 0.36 |  |  |  | 10.81 | 0.004 |  |  |  |
| Group x FA | 0.22 | 0.65 |  |  |  | 13.27 | 0.002 |  |  |  |
|  |  |  | ESC/MEM | 0.42 (0.18) | 0.03 |  |  | ESC/MEM | 0.76 (0.21) | 0.002 |
|  |  |  | ESC/PBO | 0.27 (0.28) | 0.34 |  |  | ESC/PBO | -0.16 (0.17) | 0.35 |
|  |  |  |  |  |  |  |  |  |  |  |
| **SLF** |  |  |  |  |  |  |  |  |  |  |
| FA | 9.61 | 0.007 |  |  |  | 1.84 | 0.19 |  |  |  |
| Group | 0.27 | 0.61 |  |  |  | 0.59 | 0.45 |  |  |  |
| Group x FA | 0.38 | 0.55 |  |  |  | 0.29 | 0.6 |  |  |  |
|  |  |  | ESC/MEM | 0.49 (0.18) | 0.02 |  |  | ESC/MEM | 0.35 (0.21) | 0.1 |
|  |  |  | ESC/PBO | 0.33 (0.19) | 0.1 |  |  | ESC/PBO | 0.17 (0.3) | 0.58 |
|  |  |  |  |  |  |  |  |  |  |  |
| **FX** |  |  |  |  |  |  |  |  |  |  |
| FA | 0.08 | 0.78 |  |  |  | 0.5 | 0.49 |  |  |  |
| Group | 0.14 | 0.71 |  |  |  | 0.04 | 0.84 |  |  |  |
| Group x FA | 0.38 | 0.55 |  |  |  | 0.02 | 0.89 |  |  |  |
|  |  |  | ESC/MEM | 0.04 (0.24) | 0.87 |  |  | ESC/MEM | 0.15 (0.23) | 0.53 |
|  |  |  | ESC/PBO | -0.18 (0.36) | 0.62 |  |  | ESC/PBO | 0.2 (0.37) | 0.59 |
|  |  |  |  |  |  |  |  |  |  |  |
|  | **F(1,18)** | ***p*-value** | **Group** | **Beta (SE)** | | ***p*-value** | | | | |
| **FX Body** |  |  |  |  |  |  |  |  |  |  |
| FA | 0.26 | 0.62 |  |  |  |  |  |  |  |  |
| Group | 0.02 | 0..88 |  |  |  |  |  |  |  |  |
| Group x FA | 0.09 | 0.77 |  |  |  |  |  |  |  |  |
|  |  |  | ESC/MEM | 0.16 (0.25) | 0.53 | | | | | |
|  |  |  | ESC/PBO | 0.06 (0.29) | 0.83 | | | | | |
|  |  |  |  |  |  |  |  |  |  |  |
| **GCC** |  |  |  |  |  |  |  |  |  |  |
| FA | 0.35 | 0.56 |  |  |  |  |  |  |  |  |
| Group | 0.03 | 0.87 |  |  |  |  |  |  |  |  |
| Group x FA | 0.07 | 0.8 |  |  |  |  |  |  |  |  |
|  |  |  | ESC/MEM | 0.16 (0.21) | 0.44 | | | | | |
|  |  |  | ESC/PBO | 0.07 (0.32) | 0.82 | | | | | |

HAMA = Hamilton Anxiety Scale; AES = Apathy Evaluation Scale.
